# Fractional Survival Functional Entropy of Engineering Systems

**DOI:** 10.3390/e24091275

**Published:** 2022-09-10

**Authors:** Ghadah Alomani, Mohamed Kayid

**Affiliations:** 1 Department of Mathematical Sciences, College of Science, Princess Nourah bint Abdulrahman University, P.O. Box 84428, Riyadh 11671, Saudi Arabia; 2 Department of Statistics and Operations Research, College of Science, King Saud University, P.O. Box 2455, Riyadh 11451, Saudi Arabia

**Keywords:** fractional generalized cumulative residual entropy, system signature, coherent system, Shannon entropy, stochastic orders

## Abstract

An alternate measure of uncertainty, termed the fractional generalized cumulative residual entropy, has been introduced in the literature. In this paper, we first investigate some variability properties this measure has and then establish its connection to other dispersion measures. Moreover, we prove under sufficient conditions that this measure preserves the location-independent riskier order. We then elaborate on the fractional survival functional entropy of coherent and mixed systems’ lifetime in the case that the component lifetimes are dependent and they have identical distributions. Finally, we give some bounds and illustrate the usefulness of the given bounds.

## 1. Introduction

The Shannon entropy is used in various scientific disciplines such as physics, chemistry, information theory, financial analysis, communications, engineering, and statistics, among others. The Shannon entropy is defined as H(X)=E[−logf(X)], where “log” denotes the natural logarithm, so that 0log0=0, and f(x) is the probability density function (PDF) of an absolutely continuous non-negative random variable (RV) X. It is well known that when the differential Shannon entropy considers a continuous complement of that for the discrete RVs, it presents various deficiencies. Researchers have found several methods to create surrogate measures of information. Rao et al. [1] defined the cumulative residual entropy (CRE) by

(1) $$E\left(X\right)=-\int_{0}^{\infty }S\left(x\right)\log S\left(x\right)dx=\int_{0}^{\infty }S\left(x\right)\Theta \left(x\right)dx,$$

where

(2) $$\Theta \left(x\right)=-\log S\left(x\right)=\int_{0}^{x}\eta \left(u\right)du,\;x>0,$$

is the cumulative hazard function and η(u)=f(u)/S(u),u>0, stands for the hazard rate function. Applications and the corresponding results of this function can be found in [2,3,4,5,6].

Di Crescenzo et al. [7] introduced the fractional generalized cumulative residual entropy (FGCRE) of *X* as a generalization of CRE defined by

(3) $$E_{\alpha }\left(X\right)=\frac{1}{\Gamma (\alpha +1)}\int_{0}^{\infty }S\left(x\right){\left[-\log S\left(x\right)\right]}^{\alpha }dx,$$

for all α≥0. We remark that if α is a positive integer, it can easily be seen that (3) becomes the measure of the generalized CRE established by Psarrakos and Navarro [8]. The GCRE is a quantity related to a non-homogeneous Poisson process and the distributions of the upper record values of a sequence of observations (see, e.g., [9]). The present paper establishes some properties of $E_{\alpha }\left(T\right)$ for coherent and mixed systems with lifetime *T* in situations where the component lifetimes are affected by each other and, furthermore, they are identically distributed. We recall that related results about the FCRE (as a special case of the FGCRE) can be seen in Alomani and Kayid [10], Kayid and Shrahili [11], and Xiong et al. [12]. The main theoretical properties of this paper are associated with the general properties of the FGCRE, which allows suitably extending the CRE function. Since the properties of this measure are similar to the CRE, thus, for an essential application of this measure, see the contribution given by Rao et al. [1], Toomaj et al. [6], and Toomaj and Atabay [13] and the references therein.

The rest of the paper is arranged as follows. Section 2 first establishes some basic properties of the FGCRE and then provides sufficient conditions by which it preserves the location-independent riskier order. In Section 3, we study the general properties of the FGCRE of coherent and mixed systems, where we assume that the component lifetimes are dependent and identically distributed, having a common distribution function. In the remainder, some bounds for the FGCRE of the systems’ lifetime are also obtained.

We shall denote by $R_{+}$ the set of absolutely continuous non-negative RVs having the support $R_{+}=\left(0,\infty \right).$

## 2. General Properties of FGCRE

It is worth pointing out that (3) is always non-negative, and it is suitable to measure either for the continuous or discrete distributions, while the Shannon entropy can be negative when the RV is absolutely continuous. Moreover, it is clear that for a degenerate distribution function $F_{X}$ for which X=a (a.s.), we have $E_{\alpha }\left(X\right)=0,$ that is the FGCRE has a standardization property. On the other hand, it has location invariance and the positive homogeneity property, that is $E_{\alpha }\left(aX+b\right)=aE_{\alpha }\left(X\right)$ for all a>0 and b∈R. The amount of the FGCRE is preserved under dispersion. This is an indication that the fractional survival functional entropy is a measure of variability, as given in Bickel and Lehmann [14]. Generally, the variance and standard deviation are commonly used measures of risk. We provide a bound for the FGCRE based on the standard deviation of an RV $X_{\alpha }$ with PDF

(4) $$f_{\alpha }\left(x\right)=\frac{1}{\Gamma (\alpha )}{\left[\Theta \left(x\right)\right]}^{\alpha -1}f\left(x\right),\;x\geq 0,$$

for all α>0 where Θ(x) is defined in (2).

**Theorem** **1.** *Let $X\in R_{+}$ with the survival function S(x) and standard deviation $\sigma (X_{\alpha })<\infty$ for all α>0. Then, under the condition that the expectation exists, we have*

$$E_{\alpha }\left(X\right)\leq \sigma \left(X_{\alpha }\right),$$

*for all α>0.*

**Proof.**  From Corollary 1 of Alomani and Kayid [10], the FGCRE can be written based on the following covariance representation:

(5) $$\frac{1}{\alpha }\;\mathrm{Cov}\left(X_{\alpha },\Theta \left(X_{\alpha }\right)\right)=E_{\alpha }\left(X\right).$$

Using the Cauchy–Schwartz inequality for (5), we obtain

$${\left[\mathrm{Cov}\left(X_{\alpha },\Theta \left(X_{\alpha }\right)\right)\right]}^{2}\leq Var\left[\Theta \left(X_{\alpha }\right)\right]Var\left(X_{\alpha }\right)=\alpha Var\left(X_{\alpha }\right),$$

where the last equality is due to $Var[\Theta \left(X_{\alpha }\right)]=\alpha$ because $\Theta (X_{\alpha })$ has a gamma distribution with the shape parameter α and scale parameter one. Therefore, this completes the proof. □

Another useful connection is between the FGCRE and the generalized Gini mean difference, defined by

$$D_{\alpha }\left(X\right)=\frac{1}{\Gamma (\alpha +1)}\int_{0}^{\infty }F^{\alpha }\left(x\right)S\left(x\right)dx,\;\alpha \geq 0.$$

Specially, when α=1, we have the well-known Gini mean difference as

$$D_{G}\left(X\right)=\int_{0}^{\infty }S\left(x\right)\left(1-S\left(x\right)\right)dx.$$

**Figure 1:**
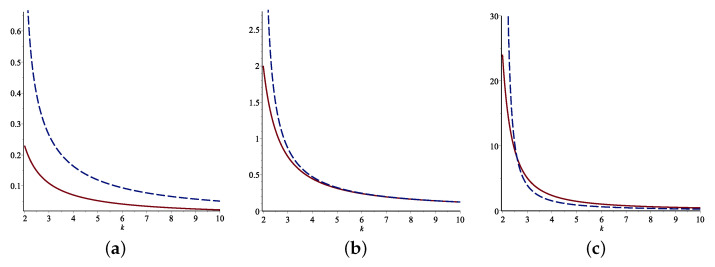

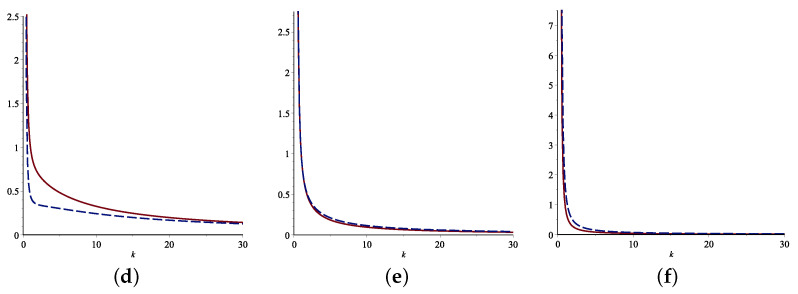
Comparisons of the standard deviation (blue line) and FGCRE (red line) for the Pareto (**top**) and Weibull (**bottom**) models for various values of α when b=1. (**a**) α=0.2; (**b**) α=1; (**c**) α=3; (**d**) α=0.2; (**e**) α=1; (**f**) α=3.

Therefore, from Theorem 3 of Alomani and Kayid [10], we have $E_{\alpha }\left(X\right)\geq D_{\alpha }\left(X\right)$ for all α≥0. Let $S^{-1}\left(u\right)=\sup\left\{x:S\left(x\right)\geq u\right\},\;0\leq u\leq 1,$ be the quantile function of *S*. If U=F(X), one can write the FGCRE as

(6) $$E_{\alpha }\left(X\right)=\int_{0}^{1}\frac{\psi_{\alpha }\left(u\right)}{f(S^{-1}\left(u\right))}du,$$

where

$$\psi_{\alpha }\left(u\right)=\frac{u{\left(-\log u\right)}^{\alpha }}{\Gamma (\alpha +1)},\;0\leq u\leq 1,$$

where $\psi_{\alpha }\left(0\right)=\psi_{\alpha }\left(1\right)=0.$ Some examples of the FGCRE and the standard deviation of $X_{\alpha }$ are given in Table 1. The FGCRE and the standard deviation are compared with respect to *k* for various values of α for some distributions. They are shown in Figure 1. Based on these graphs, the relationship that the FGCRE has with the standard deviation of $X_{\alpha }$ is detected.

Here, we establish that the fractional generalized cumulative residual entropy preserves the well-known dispersive and location-independent riskier order. First, we recall the mentioned notions.

**Definition** **1.** *Let $X_{1}\in R_{+}$ and $X_{2}\in R_{+}$ with the CDFs $F_{1}$ and $F_{2}$ and the survival functions $S_{1}$ and $S_{2},$ respectively. Then, we say that:*

*1.*  *$X_{1}$ is smaller than $X_{2}$ in the dispersive order (denoted by $X_{1}\leq_{d}X_{2}$) if $S_{1}^{-1}\left(u\right)-S_{1}^{-1}\left(v\right)\leq S_{2}^{-1}\left(u\right)-S_{2}^{-1}\left(v\right),\;0<u\leq v<1$.*
*2.*  *$X_{1}$ is smaller than $X_{2}$ in the location-independent riskier order (denoted by $X_{1}\leq_{lir}X_{2}$) if $\int_{0}^{F_{1}^{-1}\left(p\right)}F_{1}\left(x\right)dx\leq \int_{0}^{F_{2}^{-1}\left(p\right)}F_{2}\left(x\right)dx,\;p\in \left(0,1\right).$*

We remark that if $X_{1}$ and $X_{2}$ are absolutely continuous with PDFs $f_{1}$ and $f_{2}$, respectively, then $X_{1}\leq_{d}X_{2}$ is equivalent to

(7) $$f_{2}\left(S_{2}^{-1}\left(v\right)\right)\leq f_{1}\left(S_{1}^{-1}\left(v\right)\right),\;0<v<1.$$

It is clear that $X_{1}\leq_{d}X_{2}$ gives $E_{\alpha }\left(X_{1}\right)\leq E_{\alpha }\left(X_{2}\right)$ due to (6). Since $X_{1}\leq_{d}X_{2}$ is a sufficient condition for $E_{\alpha }\left(X_{1}\right)\leq E_{\alpha }\left(X_{2}\right),$ one can define the following order.

**Definition** **2.** *Let $X_{1},X_{2}\in R_{+}.$ We say that $X_{1}$ is said to be smaller than $X_{2}$ in the fractional generalized cumulative residual entropy order (denoted by $X_{1}\leq_{FGCRE}X_{2}$) if $E_{\alpha }\left(X_{1}\right)\leq E_{\alpha }\left(X_{2}\right)$ for all α≥0.*

We should note that if $X_{1}{=}_{FGCRE}X_{2},$ then it does not necessarily mean that $X_{1}$ and $X_{2}$ are identically distributed. For a strictly increasing function ϕ, let us consider $X_{2}=\phi \left(X_{1}\right).$ Then, recalling Relation (14) of Kayid and Shrahili [11], one can write

(8) $$E_{\alpha }\left(X_{2}\right)=\frac{1}{\Gamma (\alpha +1)}\int_{0}^{\infty }\phi^{\prime }\left(u\right)S_{1}\left(u\right){\left[-\log S_{1}\left(u\right)\right]}^{\alpha }du,$$

for all α≥0. Therefore, if $\phi^{\prime }\left(u\right)\geq 1$, then $X_{1}\leq_{FGCRE}X_{2}$, which is similar to Theorem 1 of Ebrahimi et al. [15]. The integrated distribution function of *H* for every RV *Z* with CDF *H* is defined by

(9) $$\Psi_{Z}\left(x\right)=\int_{0}^{x}H\left(t\right)dt,\;x>0.$$

It was proven by Landsberger and Meilijson (1994) that

(10) $$X_{1}\leq_{lir}X_{2}⟺\Psi_{X_{2}}^{-1}\left(x\right)-\Psi_{X_{1}}^{-1}\left(x\right)\;\mathrm{is}\;\mathrm{increasing}\;\mathrm{in}\;x>0.$$

We now state and prove that $E_{\alpha }\left(X_{1}\right)\leq E_{\alpha }\left(X_{2}\right)$ is a necessary condition for the location-independent riskier order $X_{1}\leq_{lir}X_{2}.$

**Theorem** **2.** *Let $X_{1},X_{2}\in R_{+}$ with the DFs $F_{1}$ and $F_{2}$ and survival functions $S_{1}$ and $S_{2},$ respectively. If $X_{1}\leq_{lir}X_{2},$ then $E_{\alpha }\left(X_{1}\right)\leq E_{\alpha }\left(X_{2}\right)$ for all 0≤α≤1.*

**Proof.**  From assumption $X_{1}\leq_{lir}X_{2}$ and, hence, Relation (10), we have

$$\frac{d}{dx}\left(\Psi_{X_{2}}^{-1}\left(x\right)-\Psi_{X_{1}}^{-1}\left(x\right)\right)=\frac{1}{F_{2}\left(\Psi_{X_{2}}^{-1}\left(x\right)\right)}-\frac{1}{F_{1}\left(\Psi_{X_{1}}^{-1}\left(x\right)\right)}\geq 0,\;\forall \;x>0.$$

This implies that

(11) $$F_{1}\left(x\right)\geq F_{2}\left(\Psi_{X_{2}}^{-1}\left(\Psi_{X_{1}}\left(x\right)\right)\right),\;\forall \;x>0.$$

To prove the assertion, we have

(12) $$\begin{array}{ccc} \int_{0}^{\infty }S_{1}\left(x\right){\left[-\log S_{1}\left(x\right)\right]}^{\alpha }dx & = & \int_{0}^{\infty }\frac{S_{1}\left(x\right){\left[-\log S_{1}\left(x\right)\right]}^{\alpha }}{F_{1}\left(x\right)}F_{1}\left(x\right)dx\\ & \leq & \int_{0}^{\infty }\frac{S_{2}\left(\Psi_{X_{2}}^{-1}\left(\Psi_{X_{1}}\left(x\right)\right)\right){\left[-\log S_{2}\left(\Psi_{X_{2}}^{-1}\left(\Psi_{X_{1}}\left(x\right)\right)\right)\right]}^{\alpha }}{F_{2}\left(\Psi_{X_{2}}^{-1}\left(\Psi_{X_{1}}\left(x\right)\right)\right)}\\ & \times & F_{1}\left(x\right)dx, \end{array}$$

where the inequality is due to $\left(1-x\right){\left(-\log\left(1-x\right)\right)}^{\alpha }/x$ being decreasing in 0≤x≤1 for all 0≤α≤1 and recalling (11). Let us set $u=\Psi_{X_{2}}^{-1}\left(\Psi_{X_{1}}\left(x\right)\right)$ and then

$$dx=\frac{F_{2}\left(u\right)}{F_{1}\left(\Psi_{X_{1}}^{-1}\left(\Psi_{X_{2}}\left(u\right)\right)\right)}du.$$

Upon using this, (12) reduces to

$$\begin{array}{ccc} & & \int_{0}^{\infty }\frac{S_{2}\left(\Psi_{X_{2}}^{-1}\left(\Psi_{X_{1}}\left(x\right)\right)\right){\left[-\log S_{2}\left(\Psi_{X_{2}}^{-1}\left(\Psi_{X_{1}}\left(x\right)\right)\right)\right]}^{\alpha }}{F_{2}\left(\Psi_{X_{2}}^{-1}\left(\Psi_{X_{1}}\left(x\right)\right)\right)}F_{1}\left(x\right)dx\\ & \leq & \int_{\Psi_{X_{2}}^{-1}\left(\Psi_{X_{1}}\left(0\right)\right)}^{\infty }\frac{S_{2}\left(u\right){\left[-\log S_{2}\left(u\right)\right]}^{\alpha }}{F_{2}\left(u\right)}\frac{F_{1}\left(\Psi_{X_{1}}^{-1}\left(\Psi_{X_{2}}\left(u\right)\right)\right)F_{2}\left(u\right)}{F_{1}\left(\Psi_{X_{1}}^{-1}\left(\Psi_{X_{2}}\left(u\right)\right)\right)}du\\ & = & \int_{0}^{\infty }S_{2}\left(u\right){\left[-\log S_{2}\left(u\right)\right]}^{\alpha }du, \end{array}$$

where the last equality is obtained by noting that $\Psi_{X_{2}}^{-1}\left(\Psi_{X_{1}}\left(0\right)\right)=0$; hence, we obtain $E_{\alpha }\left(X_{1}\right)\leq E_{\alpha }\left(X_{2}\right)$ for all 0≤α≤1 by recalling (3). This completes the proof. □

## 3. Application to Coherent and Mixed Systems

In this section, we establish some coherent and mixed systems’ properties. The *k*-out-of-*n* system is a coherent system where the system fails when the *k*-th component failure occurs. A stochastic mixture of coherent systems is termed the mixed system (see, e.g., Samaniego [16]). If *T* stands for the mixed system’s lifetime with *n* independent and identically distributed (iid) component lifetimes $X_{1},\cdots ,X_{n}$ having absolutely continuous CDF F, the survival or reliability function of the mixed system is

(13) $$\begin{array}{c} S_{T}\left(t\right)=P\left(T>t\right)=\sum_{i=1}^{n}p_{i}S_{i:n}\left(t\right), \end{array}$$

where Si:n(t)=∑j=0i−1nj[F(t)]j[S(t)]n−j for i=1,⋯,n are the reliability functions of $X_{1:n},\cdots ,X_{n:n}$. In the literature, the vector of coefficients **p** = $\left(p_{1},\cdots ,p_{n}\right)$ in $S_{T}\left(t\right)$ is denominated as the *system signature*, where $p_{i}=P\left(T=X_{i:n}\right).$ It should be noted that the elements $p_{1},\cdots ,p_{n}$ are non-negative real numbers between [0,1], where the parent CDF *F* plays no role and the identity $\sum_{i=1}^{n}p_{i}=1$ holds.

Here, we first give an expression for the FGCRE of a mixed system with the system signature $p=(p_{1},\cdots ,p_{n})$ consisting of *n* iid component lifetimes $X_{1},\cdots ,X_{n}$ with CDF *F* and PDF f. It is well known that the probability integral transformation $U_{i}=S\left(X_{i}\right)$ is uniformly distributed in [0,1]. Thus, the CDF of $U_{i:n}=S\left(X_{i:n}\right)$ is

(14) Gi:n(u)=∑j=0i−1nj(1−u)jun−j,0≤u≤1,

for i=1,⋯,n. Therefore, the CDF of the probability integral transformation V=S(T) is

(15) $$\begin{array}{c} G_{V}\left(v\right)=\sum_{i=1}^{n}p_{i}G_{i:n}\left(v\right),\;0\leq v\leq 1. \end{array}$$

Recalling (1) and the earlier stated transforms, we have $S_{T}\left(t\right)=G_{V}\left(S\left(t\right)\right)$ and

(16) $$\begin{array}{c} E_{\alpha }\left(T\right)=\frac{1}{\Gamma (\alpha +1)}\int_{0}^{\infty }S_{T}\left(t\right){\left[-\log S_{T}\left(t\right)\right]}^{\alpha }dx=\int_{0}^{1}\frac{\psi_{\alpha }\left(G_{V}\left(v\right)\right)}{f(S^{-1}\left(v\right))}dv, \end{array}$$

where $\psi_{\alpha }\left(v\right)=\frac{v{\left(-\log v\right)}^{\alpha }}{\Gamma (\alpha +1)},\;0\leq v\leq 1,$ for all α≥0.

It was proven by Navarro et al. [17] that $S_{T}\left(t\right)$ with dependent and identically distributed (did) component lifetimes can be written as

(17) $$\begin{array}{c} S_{T}\left(t\right)=h\left(S\left(t\right)\right),\;t>0, \end{array}$$

where *h* is a distortion function in the sense that it is an increasing continuous function in [0,1] such that h(0)=0 and h(1)=1 and *S* is the common baseline reliability function of the components. We remark that in the distortion function h, the CDF plays no role, and it only depends on the structure function and on the copula of the random vector $(X_{1}\cdots ,X_{n}).$ In particular, if the component lifetimes $\left(X_{1}\cdots ,X_{n}\right)$ are exchangeable (i.e., every permutation of the vector has the same joint distribution), then

(18) $$\begin{array}{c} h\left(v\right)=\sum_{i=1}^{n}a_{i}J\left(v_{i}\right), \end{array}$$

where $v_{i}=\left(u_{1},\cdots ,u_{n}\right)$ with $u_{1}=\cdots =u_{i}=v$ and $u_{i+1}=\cdots =u_{n}=1$ and *J* is the exchangeable survival copula of $(X_{1},\cdots ,X_{n}).$ The coefficients $\left(a_{1},\cdots ,a_{n}\right)$ in (18) are the minimal signature the system has. Specially, if the component lifetimes are iid, then (see, e.g., [3])

(19) $$\begin{array}{c} h\left(v\right)=G_{V}\left(v\right)=\sum_{i=1}^{n}a_{i}v^{i}. \end{array}$$

Therefore, the representation (16) can be generalized to the mixed systems with did components; hence, from (17), one can write

(20) $$\begin{array}{c} E_{\alpha }\left(T\right)=\frac{1}{\Gamma (\alpha +1)}\int_{0}^{\infty }S_{T}\left(t\right){\left[-\log S_{T}\left(t\right)\right]}^{\alpha }dx=\int_{0}^{1}\frac{\psi_{\alpha }\left(h\left(v\right)\right)}{f(S^{-1}\left(v\right))}dv, \end{array}$$

for all α≥0. As an application of Equations (16) and (20), consider the following example.

**Example** **1.** *Consider a coherent system with lifetime $T=\max\{\min\left\{X_{1},X_{2}\right\},\min\left\{X_{3},X_{4}\right\}\}$ consisting of n=4 iid components with S(t)=exp(−t/λ) for t≥0 and λ≥0. The signature is p=(0,2/3,1/3,0), and its minimal signature is* ***a*** *= (0,2,0,−1). It is clear that $f(S^{-1}\left(v\right))=v/\lambda$; thus, we have*

$$E_{\alpha }\left(T\right)=\frac{\lambda }{\Gamma (\alpha +1)}\int_{0}^{1}\left(2v-v^{3}\right){\left(-\log(2v^{2}-v^{4})\right)}^{\alpha }dv,$$

**Figure 2:**
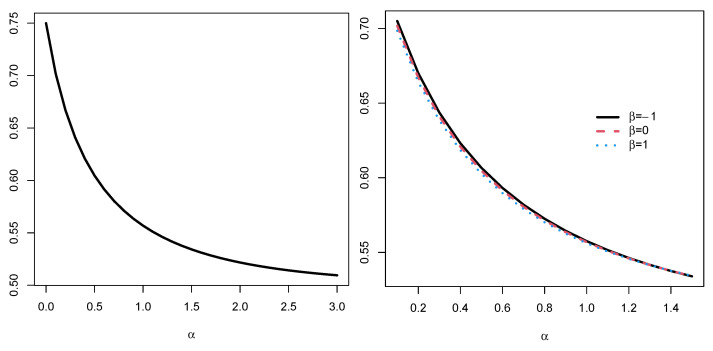
The plot of $E_{\alpha }\left(T\right)$ with iid (**left panel**) and did (**right panel**) with respect to α in Example 1.

*for all α≥0. Clearly, it can be seen that the FGCRE is increasing with respect to λ in the sense that the variability of the system’s lifetime increases with increasing the parameter λ; however, it is decreasing with respect to the parameter α, as shown in Figure 2 (left panel). Now, suppose the component lifetimes share the Farlie–Gumbel–Morgenstern copula as*

$$\begin{array}{c} J\left(u_{1},u_{2},u_{3},u_{4}\right)=u_{1}u_{2}u_{3}u_{4}\left(1+\beta \left(1-u_{1}\right)\left(1-u_{2}\right)\left(1-u_{3}\right)\left(1-u_{4}\right)\right), \end{array}$$

*for β∈[−1,1]. The reliability function of the system is $S_{T}\left(t\right)=2S_{1:2}\left(t\right)-S_{1:4}\left(t\right)=h\left(S\left(t\right)\right),$ where $h\left(v\right)=2J\left(v,v,1,1\right)-J\left(v,v,v,v\right)=2v^{2}-v^{4}\left(1+\beta {\left(1-v\right)}^{4}\right)$. Consider the case when the components are exponential. Then, the FGCRE is*

$$\begin{array}{c} E_{\alpha }\left(T\right)=\frac{\mu }{\Gamma (\alpha +1)}\int_{0}^{1}\left(2v-v^{3}\left(1+\beta {\left(1-v\right)}^{4}\right)\right){\left(-\log\left(2v^{2}-v^{4}\left(1+\beta {\left(1-v\right)}^{4}\right)\right)\right)}^{\alpha }dv. \end{array}$$

*It is hard to obtain a closed-form expression for $E_{\alpha }\left(T\right)$, and so, we compute it numerically. One can see in Figure 2 (right panel) that $E_{\alpha }\left(T\right)$ decreases when the dependence parameter β changes in [−1,1] for all values of α.*

We recall that the minimal signatures of the systems with 1–5 components were computed in [3], and so, one can compute the values of $E_{\alpha }\left(T\right)$ numerically for all α≥0. For instance, for various values of α, we give the FGCRE of these systems with 1–4 iid exponential components in Table 2. The values of $E_{\alpha }\left(T\right)$ and the respective standard deviations of $T_{\alpha }$ for some values of α are given in Table 2. An interesting result is to compare the FGCRE of two mixed systems with the same structure having did component lifetimes by using Equation (20), which is stated in the next theorem.

**Theorem** **3.** *Let us assume that $T^{X_{1}}$ and $T^{X_{2}}$ are the lifetimes of two mixed systems having the same structure consisting of n did component lifetimes with the same copula and DFs $F_{1}$ and $F_{2}$ and PDFs $f_{1}$ and $f_{2},$ respectively:*

*(i)*  *If $X_{1}\leq_{d}X_{2}$, then $T^{X_{1}}\leq_{FGCRE}T^{X_{2}}$.*
*(ii)*  *If $X_{1}\leq_{FGCRE}X_{2}$ and for all α≥0,*

(21) $$\underset{v\in A_{1}}{\inf}\frac{\psi_{\alpha }\left(h\left(v\right)\right)}{\psi_{\alpha }\left(v\right)}\geq \underset{v\in A_{2}}{\sup}\frac{\psi_{\alpha }\left(h\left(v\right)\right)}{\psi_{\alpha }\left(v\right)},$$

*for $A_{1}=\left\{v\in \left[0,1\right]:f_{1}\left(S_{1}^{-1}\left(v\right)\right)>f_{2}\left(S_{2}^{-1}\left(v\right)\right)\right\}$, $A_{2}=\left\{v\in \left[0,1\right]:f_{1}\left(S_{1}^{-1}\left(v\right)\right)\leq f_{2}\left(S_{2}^{-1}\left(v\right)\right)\right\}$, then $T^{X_{1}}\leq_{FGCRE}T^{X_{2}}$.*

**Proof.**  (i) The structure function of the systems is the same, and also, they have the same copula. This implies that they have the same distortion function *h*. On the other hand, from assumption $X_{1}\leq_{d}X_{2}$ and, hence, from (7), it holds that

$$\frac{\psi_{\alpha }\left(h\left(v\right)\right)}{f_{1}\left(S_{1}^{-1}\left(v\right)\right)}\leq \frac{\psi_{\alpha }\left(h\left(v\right)\right)}{f_{2}\left(S_{2}^{-1}\left(v\right)\right)},\;$$

for all 0<v<1, where $\psi_{\alpha }\left(h\left(v\right)\right)\geq 0$ for all α≥0. Hence, Expression (20) completes the proof. Part (ii) can be proven in a similar manner as Theorem 1 of [6], and hence, we omit it. □

Due to the assumptions of the above theorem and since *h* is strictly increasing in (0,1), it was proven in [17] that $X_{1}\leq_{d}X_{2}$ coincides with $T^{X_{1}}\leq_{d}T^{X_{2}}$. Moreover, when the component lifetimes are iid, because of the polynomial property, then *h* is always strictly increasing in (0,1), and so, this equivalence holds.

**Example** **2.** *Assume a coherent system with lifetime $T^{X}=\min\left\{X_{1},\max\left\{X_{2},X_{3}\right\}\right\}$ where $X_{1},X_{2},X_{3}$ are iid from the CDF:*

(22) $$F_{X}\left(t\right)=1-e^{-2t},\;t>0,$$

*and let $T^{Z}=\min\left\{Z_{1},\max\left\{Z_{2},Z_{3}\right\}\right\}$ be another coherent system with the iid component lifetimes $Z_{1},Z_{2},Z_{3}$ having the common CDF:*

(23) $$F_{Z}\left(t\right)=1-e^{-t},\;t>0.$$

*The minimal signature of the system is p=(0,2,−1). The FGCREs of these lifetimes are $E_{\alpha }\left(X\right)=1/2$ and $E_{\alpha }\left(Z\right)=1,$ respectively. Thus, we obtain $X\leq_{FGCRE}Z$. Moreover, it can be seen that $A_{1}=\left[0,1\right)$ and $A_{2}=\left\{1\right\}.$ Since*

$$h\left(v\right)=G_{V}\left(v\right)=2v^{2}-v^{3},\;0\leq v\leq 1,$$

**Figure 3:**
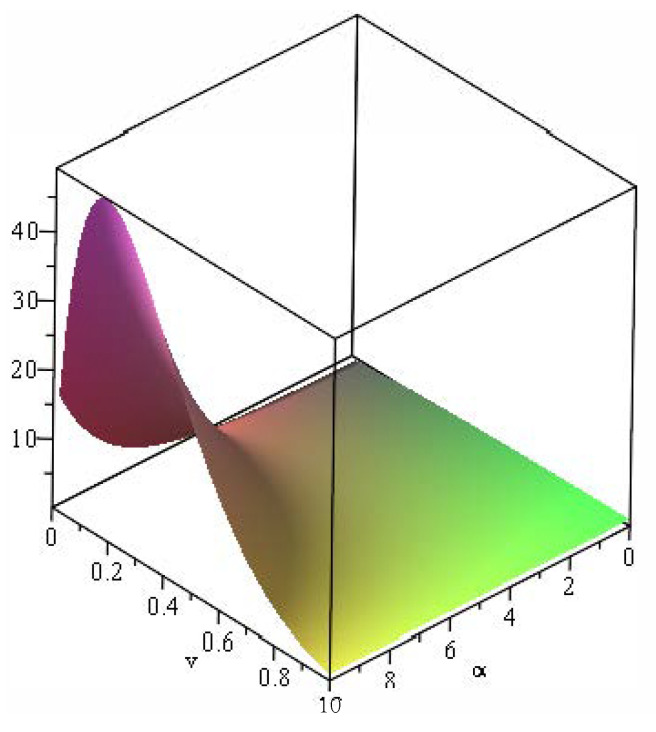
The plot of function $\psi_{\alpha }\left(h\left(v\right)\right)/\psi_{\alpha }\left(v\right)$ with respect to α and *v* in Example 2.

*and due to Figure 3, one can obtain*

$$\underset{v\in A_{1}}{\inf}\frac{\psi_{\alpha }\left(h\left(v\right)\right)}{\psi_{\alpha }\left(v\right)}=\underset{v\in A_{2}}{\sup}\frac{\psi_{\alpha }\left(h\left(v\right)\right)}{\psi_{\alpha }\left(v\right)}=0,$$

*for all α≥0 and 0≤v≤1. Thus, Part (ii) of Theorem 3 yields $T^{X}\leq_{FGCRE}T^{Z}$.*

The preservation of mixed systems under the location-independent riskier order is established for lifetimes of coherent and mixed systems under some conditions on the distortion functions in the next theorem.

**Theorem** **4.** *Under the assumption of Theorem 3, if $X_{1}\leq_{lir}X_{2}$ and*

(24) $$\frac{h\left(1-x\right){\left(-\log h\left(1-x\right)\right)}^{\alpha }}{x},\;0\leq x\leq 1,$$

*is decreasing in x for all α≥0, then $T^{X_{1}}\leq_{FGCRE}T^{X_{2}}$.*

**Proof.**  Assumption $X_{1}\leq_{lir}X_{2}$ yields (11). From this and by noting that the function (24) is decreasing in *x* for all 0≤α≤1,

$$\begin{array}{ccc} \int_{0}^{\infty }S_{T^{X_{1}}}\left(x\right){\left[-\log S_{T^{X_{1}}}\left(x\right)\right]}^{\alpha }dx & = & \int_{0}^{\infty }\frac{S_{T^{X_{1}}}\left(x\right){\left[-\log S_{T^{X_{1}}}\left(x\right)\right]}^{\alpha }}{F_{1}\left(x\right)}F_{1}\left(x\right)dx\\ & = & \int_{0}^{\infty }\frac{h\left(S_{X_{1}}\left(x\right)\right){\left[-\log h\left(S_{X_{1}}\left(x\right)\right)\right]}^{\alpha }}{F_{1}\left(x\right)}F_{1}\left(x\right)dx\\ & \leq & \int_{0}^{\infty }\frac{h(S_{X_{2}}\left(\Psi_{X_{2}}^{-1}\left(\Psi_{X_{1}}\left(x\right)\right)\right))L\left(x,\alpha \right)}{F_{2}\left(\Psi_{X_{2}}^{-1}\left(\Psi_{X_{1}}\left(x\right)\right)\right)}F_{1}\left(x\right)dx, \end{array}$$

where $L\left(x,\alpha \right)={\left[-\log h\left(S_{X_{2}}\left(\Psi_{X_{2}}^{-1}\left(\Psi_{X_{1}}\left(x\right)\right)\right)\right)\right]}^{\alpha }$. In the spirit of the proof of Theorem 2 and letting $u=\Psi_{X_{2}}^{-1}\left(\Psi_{X_{1}}\left(x\right)\right)$, we have

$$\begin{array}{ccc} & & \int_{\Psi_{X_{2}}^{-1}\left(\Psi_{X_{1}}\left(0\right)\right)}^{\infty }\frac{h\left(S_{X_{2}}\left(u\right)\right){\left[-\log h\left(S_{X_{2}}\left(u\right)\right)\right]}^{\alpha }}{F_{2}\left(u\right)}\frac{F_{1}\left(\Psi_{X_{1}}^{-1}\left(\Psi_{X_{2}}\left(u\right)\right)\right)F_{2}\left(u\right)}{F_{1}\left(\Psi_{X_{1}}^{-1}\left(\Psi_{X_{2}}\left(u\right)\right)\right)}du\\ & = & \int_{\Psi_{X_{2}}^{-1}\left(\Psi_{X_{1}}\left(0\right)\right)}^{\infty }h\left(S_{X_{2}}\left(u\right)\right){\left[-\log h\left(S_{X_{2}}\left(u\right)\right)\right]}^{\alpha }du\\ & = & \int_{0}^{\infty }h\left(S_{X_{2}}\left(u\right)\right){\left[-\log h\left(S_{X_{2}}\left(u\right)\right)\right]}^{\alpha }du\\ & = & \int_{0}^{\infty }S_{T^{X_{2}}}\left(x\right){\left[-\log S_{T^{X_{2}}}\left(x\right)\right]}^{\alpha }dx, \end{array}$$

and hence, we obtain $E_{\alpha }\left(T^{X_{1}}\right)\leq E_{\alpha }\left(T^{X_{2}}\right)$ for all α≥0. This completes the proof of the theorem. □

As an application of the above theorem, consider the next example.

**Example** **3.** *Let $T^{X}=\max\left\{X_{1},\min\left\{X_{2},X_{3},X_{4}\right\}\right\}$ be the lifetime a coherent system has, where $X_{1},X_{2},X_{3},X_{4}$ are the lifetimes of its components, with CDF*

(25) $$F_{X}\left(t\right)=1-{\left(\frac{1}{1+t}\right)}^{3},\;t>0.$$

*In this case, we have $F_{X}^{-1}\left(p\right)={\left(1-p\right)}^{-1/3}-1,\;0\leq p\leq 1$, and thus, we obtain*

$$M_{X}\left(p\right)=\int_{0}^{F_{X}^{-1}\left(p\right)}F_{X}\left(x\right)dx=\frac{1}{\sqrt[{3}]{1-p}}+\frac{1}{2}\left[\sqrt[{3}]{{\left(1-p\right)}^{2}}-3\right],\;0\leq p\leq 1.$$

*Moreover, let $T^{Z}=\max\left\{Z_{1},\min\left\{Z_{2},Z_{3},Z_{4}\right\}\right\}$ be the lifetime of the coherent system with component lifetimes $Z_{1},Z_{2},Z_{3},Z_{4}$, which are iid, and the common CDF*

(26) $$F_{Z}\left(t\right)=1-{\left(\frac{1}{1+t}\right)}^{2},\;t>0,$$

*where $F_{Z}^{-1}\left(p\right)={\left(1-p\right)}^{-1/2}-1,\;0\leq p\leq 1,$ and so, we obtain*

$$M_{Z}\left(p\right)=\int_{0}^{F_{Z}^{-1}\left(p\right)}F_{Z}\left(x\right)dx=\frac{1}{\sqrt{1-p}}+\frac{1}{2}\left[\sqrt{1-p}-4\right],\;0\leq p\leq 1.$$

**Figure 4:**
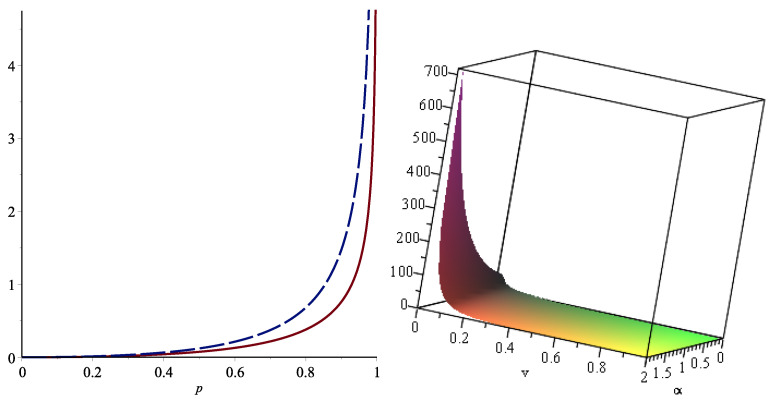
The plot $M_{X}\left(p\right)$ (solid line) and $M_{Z}\left(p\right)$ (dashed line) in the **left panel** and the function (24) with respect to α and *v* in the **right panel** in Example 3.

*Moreover, the minimal signature of the system is a=(1,0,1,−1). In Figure 4, we plot the functions $M_{X}\left(p\right)$ (solid line) and $M_{Z}\left(p\right)$ (dashed line), where one can see that $M_{X}\left(p\right)\leq M_{Z}\left(p\right)$ for all 0≤p≤1; thus, this results in $X_{1}\leq_{lir}X_{2}.$ Since the function (24) is decreasing in this case (right panel), Theorem 4 yields $T^{X}\leq_{FGCRE}T^{Z}$.*

### FGCRE of the Systems and Bounds

Hereafter, using the results obtained in the previous section, we obtain some bounds for the FGCRE of mixed systems. We point out that, in general, it is difficult or, in some cases, impossible to evaluate the FGCRE of the system’s lifetime when the system has a complicated structure function or its components are large. Therefore, it is very useful to provide bounds for the FGCRE of the system’s lifetime to approximate its behavior. In the next theorem, we first provide bounds for the FGCRE of the system on the basis of the common FGCRE of the components and then obtain the bounds in terms of the bounded PDF and the underlying distortion function.

**Theorem** **5.** *Let T represent the lifetime a mixed system has with i.d. component lifetimes $X_{1},\cdots ,X_{n}$, and let h be the associated distortion function:*

*(a)*  *If we denote $\xi_{1,\alpha }=\inf_{v\in (0,1)}\frac{\psi_{\alpha }\left(h\left(v\right)\right)}{\psi_{\alpha }\left(v\right)}$, $\xi_{2,\alpha }=\sup_{v\in (0,1)}\frac{\psi_{\alpha }\left(h\left(v\right)\right)}{\psi_{\alpha }\left(v\right)}$, and*
$$\psi_{\alpha }\left(u\right)=u{\left(-\log\left(u\right)\right)}^{\alpha }/\Gamma \left(\alpha +1\right),$$
  *then $\xi_{1,\alpha }E\left(X\right)\leq E_{\alpha }\left(T\right)\leq \xi_{2,\alpha }E\left(X\right)$ for all α≥0.*
*(b)*  *If $l=\inf_{x\in D}f\left(x\right)$ and $L=\sup_{x\in D}f\left(x\right)$, where D is the support of f, then*
(27) $$\begin{array}{c} \frac{1}{L}I_{h,\alpha }\leq E_{\alpha }\left(T\right)\leq \frac{1}{l}I_{h,\alpha }, \end{array}$$
  *where $I_{h,\alpha }=\int_{0}^{1}\psi_{\alpha }\left(h\left(v\right)\right)dv$ and $\psi_{\alpha }\left(u\right)=u{\left(-\log\left(u\right)\right)}^{\alpha }/\Gamma \left(\alpha +1\right)$.*

**Proof.**  (a) The upper bound can be obtained from (20) as follows:

$$\begin{array}{cc} E_{\alpha }\left(T\right)\; & =\int_{0}^{1}\frac{\psi_{\alpha }\left(h\left(v\right)\right)}{f(S^{-1}\left(v\right))}dv=\int_{0}^{1}\frac{\psi_{\alpha }\left(h\left(v\right)\right)}{\psi_{\alpha }\left(v\right)}\frac{\psi_{\alpha }\left(v\right)}{f(S^{-1}\left(v\right))}dv\\ \; & \leq \underset{v\in (0,1)}{\sup}\frac{\psi_{\alpha }\left(h\left(v\right)\right)}{\psi_{\alpha }\left(v\right)}\int_{0}^{1}\frac{\psi_{\alpha }\left(v\right)}{f(S^{-1}\left(v\right))}dv=\xi_{2,\alpha }E\left(X\right). \end{array}$$

In a similar manner, one can obtain the lower bound.

(b) By noting that $l\leq f(S^{-1}\left(v\right))\leq L,\;0<v<1$, from (16), we have

$$\begin{array}{cc} E_{\alpha }\left(T\right) & =\int_{0}^{1}\frac{\psi_{\alpha }\left(h\left(v\right)\right)}{f(S^{-1}\left(v\right))}dv\geq \frac{1}{L}\int_{0}^{1}\psi_{\alpha }\left(h\left(v\right)\right)dv. \end{array}$$

Similarly, the upper bound can be derived. □

It is worth pointing out that $I_{h,\alpha }$ can be written as follows:

$$\begin{array}{c} I_{h,\alpha }=\int_{0}^{1}\psi_{\alpha }\left(h\left(v\right)\right)dv=\int_{0}^{1}\psi_{\alpha }\left(h\left(1-v\right)\right)dv=E_{\alpha }\left(T_{U}\right). \end{array}$$

We remark that $T_{U}=F\left(T\right)$ denotes the system’s lifetime with the same distortion function of *T* and the same reliability copula *J* consisting of *n* did component lifetimes, which is uniformly distributed in [0,1]. Therefore, one can write $I_{h,\alpha }=E_{\alpha }\left(V\right)$ such that V=S(T). $I_{h,\alpha }$ depends only on the system structure and reliability copula. Moreover, it depends only on the system signature when the component lifetimes are iid. It is evident that for l=0, there is no upper bound, and if L=∞, then there is no lower bound.

**Example** **4.** *Recalling Example 1, let us assume that the components of the system are iid having a reliability function:*

$$S\left(x\right)={\left(\frac{b}{b+x}\right)}^{k},\;x\geq 0,$$

*as shown in Table 1. In this case, l=0 and $L=kb^{k}$. Therefore, $E_{\alpha }\left(T\right)\geq kb^{k}I_{h,\alpha },$ where*

$$I_{h,\alpha }=\frac{1}{\Gamma (\alpha +1)}\int_{0}^{1}\left(2v^{2}-v^{4}\right){\left(-\log(2v^{2}-v^{4})\right)}^{\alpha }dv.$$

*For example, for some values of α, we have $I_{h,0.5}=0.2794,I_{h,1}=0.1993,I_{h,1.5}=0.1508,I_{h,2}=0.1174,$ where is decreasing in α. Moreover, Part (a) of Theorem 5 gives the upper bound as $E_{\alpha }\left(T\right)\leq \xi_{2,\alpha }E\left(X\right)=\frac{\alpha bk^{\alpha }\xi_{2,\alpha }}{{\left(k-1\right)}^{\alpha +1}}$ for all α whenever k>1.*

In the next corollary, we show that the lower bound in Part (b) of Theorem 5 $\xi_{1,\alpha }=0$ for every coherent system where the lifetimes of its components are iid, and this does not remain valid for mixed systems. To this aim, if (5/8,1/8,1/8,1/8) is the signature vector of a mixed system, then it is easy to see that $\xi_{1,1}=1/2$ and $\xi_{2,1}=5/2$, which means that this is not true for all α≥0.

**Corollary** **1.** *In Part (b) of Theorem 5, the lower bound $\xi_{1,\alpha }$ is zero for all the mixed systems with iid components and signature $\left(p_{1},\cdots ,p_{n}\right)$ satisfying $p_{1}=0$ or $p_{n}=0$. Specifically, it is zero for all the coherent systems with n>1 iid components.*

**Proof.**  The proof is analogous to the proof of Proposition 3 of [6]. □

At the end of this section, under sufficient conditions on the mean residual lifetime (MRL) function of the common CDF, we establish bounds for the FGCRE. If $X_{t}=\left[X-t|X>t\right],\;t\geq 0,$ denotes the life length of a system with age t, then the mean residual life (MRL) function of *X* is

(28) $$m\left(t\right)=E\left[X-t|X>t\right]=\left\{\begin{array}{ccc} \int_{t}^{\infty }\frac{S(x)}{S(t)}\;dx,\; & \;\; & \;t>0\\ 0,\; & \;\; & \;t\leq 0 \end{array}.\right.$$

Now, we state the following theorem.

**Theorem** **6.** *Under the conditions of Theorem 5, it holds that:*

*(a)*  *If X is the DMRL and*
(29) $$\underset{u\in (0,v]}{\sup}\frac{h(u)}{u}\leq \frac{h^{2}\left(u\right)}{v^{2}h^{\prime }\left(v\right)},\;\mathrm{for}\;\mathrm{all}\;v\in \left(0,1\right),$$
  *then $E_{\alpha }\left(T\right)\leq E\left(T\right),$ for all α≥0.*
*(b)*  *If X is the IMRL and*
$$\underset{u\in (0,v]}{\inf}\frac{h(u)}{u}\geq \frac{h^{2}\left(u\right)}{v^{2}h^{\prime }\left(v\right)},\;\mathrm{for}\;\mathrm{all}\;v\in \left(0,1\right),$$
  *then $E_{\alpha }\left(T\right)\geq E\left(T\right),$ for all α≥0.*

**Proof.**  (a) We just prove Case (a); Case (b) can be obtained similarly. From the assumption that *X* is the DMRL and the condition (29) holds, then *T* is the DMRL due to Theorem 2.1 of Navarro [18]. Now, the proof is easily obtained from Theorem 7 of Kayid and Shrahili [11] as follows:

$$E_{\alpha }\left(T\right)=\int_{0}^{\infty }m_{T}\left(t\right)f_{T_{\alpha }}\left(t\right)dt\leq m_{T}\left(0\right)\int_{0}^{\infty }f_{T_{\alpha }}\left(t\right)dt=E\left(T\right),$$

and this completes the proof. □

The above theorem can be applied as follows:

**Example** **5.** *Assume the coherent system with a lifetime:*

$$T=\min\{\max\left\{X_{1},X_{2},X_{3}\right\},\max\left\{X_{2},X_{3},X_{4}\right\}\},$$

*consisting of n=4 iid component lifetimes having the common exponential distribution, which is both the IMRL and the DMRL. The minimal signature is* ***a*** *= (0,2,−2,1), and hence, its reliability function is $S_{T}\left(t\right)=h\left(S\left(t\right)\right)$, where $h\left(v\right)=2v-2v^{3}+v^{4},\;0\leq v\leq 1.$ Navarro [18] showed that*

$$\underset{u\in (0,v]}{\sup}\frac{h(u)}{u}=2\leq \frac{h^{2}\left(u\right)}{v^{2}h^{\prime }\left(v\right)},$$

*for all u∈(0,v]. Therefore, T is the DMRL, and so, Theorem 6 implies that $E_{\alpha }\left(T\right)\leq E\left(T\right)$ for all α≥0.*

## Figures and Tables

**Table 1:** The FGCRE and the standard deviation of statistical models.

Distribution	S(x)	Support	$E_{\alpha }\left(X\right)$	$\sigma (X_{\alpha })$
Uniform	$\frac{b-x}{b}$	0≤x≤b	$\frac{b}{2^{\alpha +1}}$	$b\sqrt{3^{-\alpha }-4^{-\alpha }}$
Pareto	${\left(\frac{b}{b+x}\right)}^{k}$	x≥0	$\frac{\alpha bk^{\alpha }}{{\left(k-1\right)}^{\alpha +1}}$, k>1	$b\sqrt{{\left(\frac{k}{k-2}\right)}^{\alpha }-{\left(\frac{k}{k-1}\right)}^{2\alpha }},k>2$
Weibull	$e^{-x^{k}}$	x≥0	$\frac{\Gamma (\alpha +\frac{1}{k})}{k\Gamma (\alpha +1)},\;k>0$	$\sqrt{\frac{\Gamma (\alpha +\frac{2}{k})}{\Gamma (\alpha )}-{\left(\frac{\Gamma (\alpha +\frac{1}{k})}{\Gamma (\alpha )}\right)}^{2}}$

**Table 2:** Comparisons of the FGCRE and standard deviation of $T_{\alpha }$ for some values of α and for the coherent systems having 1–4 iid components from the common standard exponential distribution.

N	*p*	*a*	$E_{0.5}\left(T\right)$	$E_{1}\left(T\right)$	$E_{2}\left(T\right)$	$\sigma (T_{0.5})$	$\sigma (T_{1})$	$\sigma (T_{2})$
1	(1)	(1)	1.0000	1.0000	1.0000	0.7071	1.0000	1.4142
2	(1, 0)	(0, 1)	0.4999	0.5000	0.5000	0.3535	0.5000	0.7071
3	(0, 1)	(2, −1)	1.2092	1.1137	1.0433	0.8641	1.1180	1.4767
4	(1, 0, 0)	(0, 0, 1)	0.3333	0.3333	0.3333	0.2357	0.3333	0.4714
5	(1/3, 2/3, 0)	(0, 2, −1)	0.6093	0.5758	0.5405	0.4327	0.5773	0.7651
6	(0, 1, 0)	(0, 3, −2)	0.6584	0.5974	0.5475	0.4720	0.6009	1.1546
7	(0, 2/3, 1/3)	(1, 1, −1)	0.9946	0.9566	0.9534	0.7062	0.9574	1.3486
8	(0, 0, 1)	(3, −3, 1)	1.3012	1.1580	1.0588	0.9400	1.1667	1.4996
9	(1, 0, 0, 0)	(0, 0, 0, 1)	0.2500	0.2500	0.2500	0.1767	0.2500	0.3535
10	(1/2, 1/2, 0, 0)	(0, 0, 2, −1)	0.3957	0.3814	0.3635	0.2803	0.3818	0.5144
11	(1/4, 3/4, 0, 0)	(0, 0, 3, −2)	0.4388	0.4064	0.3742	0.3127	0.4082	0.4223
12	(1/4, 7/12, 1/6, 0)	(0, 1, 1, −1)	0.5312	0.5061	0.4871	0.3770	0.5069	0.6890
13	(1/4, 1/4, 1/2, 0)	(0, 3, −3, 1)	0.6756	0.6255	0.5680	0.4813	0.6291	0.8055
14	(0, 1, 0, 0)	(0, 0, 4, −3)	0.4582	0.4139	0.3765	0.3288	0.4166	0.9162
15	(0, 5/6, 1/6, 0)	(0, 1, 2, −2)	0.5384	0.4984	0.4736	0.3845	0.5000	0.9171
16, 17	(0, 2/3, 1/3, 0)	(0, 2, 0, −1)	0.6046	0.5568	0.5216	0.4320	0.5590	0.7383
18, 19	(0, 1/2, 1/2, 0)	(0, 3, −2, 0)	0.6584	0.5974	0.5475	0.4720	0.6009	1.1546
20, 21	(0, 1/3, 2/3, 0)	(0, 4, −4, 1)	0.7001	0.6238	0.5609	0.5044	0.6291	0.7952
22	(0, 1/6, 5/6, 0)	(0, 5, −6, 2)	0.5609	0.6385	0.5668	0.5281	0.6455	1.4509
23	(0, 0, 1, 0)	(0, 6, −8, 3)	0.7407	0.6431	0.5683	0.5391	0.6508	2.2080
24	(0, 1/2, 1/4, 1/4)	(1, 0, 1, −1)	0.9724	0.9607	0.9752	0.6886	0.9610	1.3794
25	(0, 1/6, 7/12, 1/4)	(1, 2, −3, 1)	1.0038	0.9446	0.9322	0.7160	0.9465	1.3189
26	(0, 0, 3/4, 1/4)	(1, 3, −5, 2)	0.9946	0.9255	0.9123	0.7121	0.9279	1.8337
27	(0, 0, 1/2, 1/2)	(2, 0, −2, 1)	1.1783	1.0793	1.0210	0.8448	1.0833	1.4446
28	(0, 0, 0, 1)	(4, −6, 4, −1)	1.3528	1.1815	1.0668	0.9847	1.1932	1.5115

## Data Availability

Not applicable.

## Abbreviations

The following abbreviations are used in this manuscript:

RV(s)	Random variable(s)
CDF	Cumulative distribution function
PDF	Probability density function
CRE	Cumulative residual entropy
FGCRE	Fractional generalized cumulative residual entropy
iid	Independent and identically distributed
did	Dependent and identically distributed
MRL	Mean residual lifetime
DMRL	Increasing mean residual lifetime
IMRL	Decreasing mean residual lifetime
